# Improving access to child health services at the community level in Zambia: a country case study on progress in child survival, 2000–2013

**DOI:** 10.1093/heapol/czw141

**Published:** 2016-10-19

**Authors:** Aaron M Kipp, Margaret Maimbolwa, Marie A Brault, Penelope Kalesha-Masumbu, Mary Katepa-Bwalya, Phanuel Habimana, Sten H Vermund, Kasonde Mwinga, Connie A Haley

**Affiliations:** 1 Vanderbilt Institute for Global Health,; 2 Department of Medicine, Vanderbilt University Medical Center, Nashville, TN, USA; 3 School of Medicine, University of Zambia, Lusaka, Zambia; 4 Department of Anthropology, University of Connecticut, Storrs, CT, USA; 5 Zambia Ministry of Community Development Mother and Child Health, Lusaka, Zambia; 6 World Health Organization/Zambia Country Office, Lusaka, Zambia; 7 World Health Organization/Regional Office for Africa, Brazzaville, Congo; 8 Department of Pediatrics, Vanderbilt University Medical Center, Nashville, TN, USA

**Keywords:** Community health workers, maternal and child health, Millennium Development Goals, qualitative research, under-five mortality, Zambia

## Abstract

Reductions in under-five mortality in Africa have not been sufficient to meet the Millennium Development Goal #4 (MDG#4) of reducing under-five mortality by two-thirds by 2015. Nevertheless, 12 African countries have met MDG#4. We undertook a four country study to examine barriers and facilitators of child survival prior to 2015, seeking to better understand variability in success across countries. The current analysis presents indicator, national document, and qualitative data from key informants and community women describing the factors that have enabled Zambia to successfully reduce under-five mortality over the last 15 years and achieve MDG#4. Results identified a Zambian national commitment to ongoing reform of national health strategic plans and efforts to ensure universal access to effective maternal, neonatal and child health (MNCH) interventions, creating an environment that has promoted child health. Zambia has also focused on bringing health services as close to the family as possible through specific community health strategies. This includes actively involving community health workers to provide health education, basic MNCH services, and linking women to health facilities, while supplementing community and health facility work with twice-yearly Child Health Weeks. External partners have contributed greatly to Zambia’s MNCH services, and their relationships with the government are generally positive. As government funding increases to sustain MNCH services, national health strategies/plans are being used to specify how partners can fill gaps in resources. Zambia’s continuing MNCH challenges include basic transportation, access-to-care, workforce shortages, and financing limitations. We highlight policies, programs, and implementation that facilitated reductions in under-five mortality in Zambia. These findings may inform how other countries in the African Region can increase progress in child survival in the post-MDG period.

Key Messages

- Zambia has demonstrated strong political will with a steady series of health sector reforms over the past 20 years that have emphasized decentralization, primary health care and positive collaborations between the government and external partners.
- Community health workers, and new cadres of community health assistants, are an important extension of health services, which has brought child health services as close to the family as possible.
- Child Health Weeks are an important platform for provision of an integrated package of MNCH services at the community level and effort should be made to sustain Child Health Weeks through new funding sources.

## Introduction

**Figure 1.**
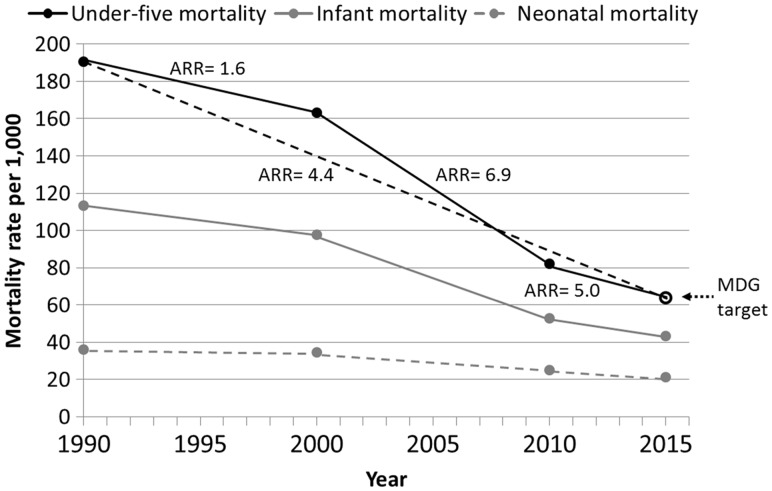
Under-five, infant, and neonatal mortality rates for Zambia in 1990, 2000, 2010 and 2015 (solid circles) with annual rates of reduction (ARR) for each period (solid and dashed lines) and the MDG target (dotted arrow, open circle). Source for data: Levels and Trends in Child Mortality: Report 2015—Estimates Developed by the United Nations Inter-agency Group for Child Mortality Estimation(UNICEF *et al.* 2015).

Under-five mortality has declined in sub-Saharan Africa from an estimated 180 deaths per 1000 live births in 1990 to 83 deaths per 1000 in 2013 (UNICEF *et al.*, 2015), yet this is insufficient to meet Millennium Development Goal (MDG) #4 of reducing under-five mortality by two-thirds between 1990 and 2015 (United Nations, 2000). Nevertheless, as of 2015, 12 African countries have met their MDG#4 goal (UNICEF *et al.*, 2015). There is, therefore, great interest in the reasons why some countries met MDG#4 while others did not. Since 2000, Zambia has made great progress in reducing under-five mortality, such that they met their MDG#4 target of 64 deaths per 1000 in 2015 (Figure 1). Infant and neonatal mortality rates have also declined, though not as rapidly as total under-five mortality. Zambia was selected for the in-depth case study presented here because of its success in reducing under-five mortality.

Zambia has successfully scaled up many maternal, neonatal, and child health (MNCH) programs and interventions [e.g. antenatal care (ANC), skilled birth attendants, immunization and infant and young child feeding practices]. Zambia has also implemented Integrated Management of Childhood Illnesses (IMCI) developed by WHO/UNICEF in the early 1990s to provide a holistic approach to managing a sick child. These efforts have likely contributed to the marked reduction in child mortality as well as other health outcomes such as childhood malnutrition and maternal mortality. Recent work in Zambia has actively sought to better understand the determinants and drivers of MNCH intervention and service coverage. Core ANC components are regularly offered to women (Bucher *et al.*, 2015), but emergency obstetric and neonatal care services are not regularly available (Owens *et al.*, 2015). Caregiver knowledge of breastfeeding and growth monitoring is good but often not practiced according to guidelines, which may be due to inadequate healthcare worker training and motivation (Charlton *et al.*, 2009; Mugala *et al.*, 2010; Katepa-Bwalya *et al.*, 2015). Other studies within Zambia have examined the feasibility and impact of community-based service delivery, demonstrating that community health workers (CHW) and coordinated community outreach efforts are feasible and effective (Yeboah-Antwi *et al.* 2010; Yeboah-Antwi *et al.*, 2014; Fiedler *et al.* 2014).

Niger, Uganda, Malawi, Ethiopia and Rwanda, have conducted case-studies to assess progress in reducing under-five mortality (Amouzou *et al.* 2012, Mbonye *et al.* 2012; Zimba *et al.* 2012; Kuruvilla *et al.* 2014); all have met MDG#4 or were on track to do so. These case studies deployed several methods, but none included qualitative information from key stakeholders or community women. Some Zambian studies have included qualitative data to better understand attitudes, behaviours, and knowledge that may further drive (or hinder) successful implementation and utilization (Charlton *et al.* 2009; Mugala *et al.* 2010; Katepa-Bwalya *et al.* 2015).

We conducted an in-depth case-study from Zambia that was part of a larger study on factors influencing progress in child survival in Africa involving countries that are both on track and not on track to meet MDG#4 (Kipp *et al.*, 2016).We used national policies and strategies, qualitative data, and quantitative indicator data to examine how changes to and implementation of key policies and strategies provide an understanding of the steady progress towards MDG#4 in Zambia, focusing on the period 2000–2013.

## Methods

The period of interest for the parent study on child survival in Africa and this case study was 2000–2013. As described below, indicator data were obtained for years closest to 2000 and 2013, while the review of national policies, key informant interviews, and focus groups with community women was conducted in 2013 and focused on more recent years.

### MNCH indicator data

Data were obtained on the core indicators monitored by Countdown to 2015. Most data were obtained from the World Bank Data Catalogue (World Bank), which is a repository of national, regional and global indicator data compiled from officially recognized sources, including national Demographic and Health Surveys (DHS) and other national surveys. Data for indicators not readily available from the World Bank Data Catalogue were obtained directly from the 2001/2002 Zambia DHS (Central Statistical Office [CSO] *et al.*, 1997) or the 2013/2014 Zambia DHS (CSO *et al.*, 2014).

Given the scope of the larger study within which this case-study is nested and recognizing that data are not always available for the exact year of interest, indicator data were obtained that most closely corresponded to the beginning of the study period in 2000 (range 1998–2003) and end of the study period in 2013 (range 2009–2014).

### Review of MNCH policies and strategies

Based on a review of the peer-reviewed literature and published global strategies related to child survival, an information abstraction guide was developed to guide the document procurement and review process for this study (Table 1). Policies and strategies pertaining to overall national health, MNCH, and those from other sectors related to MNCH (e.g., education, water and sanitation, and agriculture and nutrition) were obtained from the WHO African Region office, the WHO country focal points for Zambia and Zambia’s Ministry of Community Development, Mother and Child Health (MCDMCH). These primary documents were reviewed according to the abstraction guide and any additional documents referenced and deemed important to complete the review were obtained from WHO or MCDMCH. The final list of reviewed documents is in Supplementary Table S1.

**Table 1.** Content areas and key questions and themes related to child survival explored during the review of national health policies and strategies, key informant interview and focus groups with community women

Content Areas	Cross-cutting questions for review of national policies and strategies	Themes explored across content areas with key informants	Themes explored across content areas with community women
Health care system Leadership and governance^a^ Accountability^a^ Policies, regulations and laws^a^ National health strategies^b^ MNCH Interventions Clinical standards and guidelines^b^ Commodities and essential medicines Financial flows and resources^b^ Effective partnerships^b^ Health Information Systems/ Monitoring and Evaluation^a^ Contextual issues^c^	What policies and strategies related to child health were in place between 2000 and 2011 (including changes during this period)? What were challenges hindering progress towards MDG#4? What were facilitators enabling progress towards MDG#4? What plans for change or improvements were either implemented after 2011 or were proposed as a measure to improve child survival going forward?	Issues related to program evaluation, access and utilization, coverage, impact, and sustainability, as appropriate Knowledge and experiences related to MNCH across the *health care continuum* (prenatal care through age 5 years) Knowledge and experiences related to MNCH across the *health system continuum* (community to tertiary hospitals)	Barriers and facilitators to accessing and utilizing MNCH services, including cultural and community factors Experiences related to MNCH across the *health care continuum* Experiences related to MNCH across the *health system continuum*

a Assessed only in the review of national policies and strategies.

b Assessed only in the review of national policies and strategies and in key informant interviews.

c Domestic and international conflict, political upheaval, environmental crises, water and sanitation, nutrition and food security, education, human rights, gender-based issues and other social determinants of health.

The abstraction guide was used to standardize abstraction and summarization of content across documents. Each document was reviewed multiple times by the same author (CAH) and as needed by a second (AMK or MAB), and information was recorded as outlined in the abstraction guide. In order to avoid biased interpretation of the information documented, the abstracted information was reported as it was stated in the original source, avoiding overstating or minimizing the original information or adding commentary not contained in the source.

### Qualitative study procedures

#### Study location and participants

Because important differences in MNCH often exist between urban and rural areas, participants for the qualitative study were included from both urban and rural areas. Southern Province was selected as the study region because its under-five mortality annual rate of reduction (ARR) was comparable to the national ARR based on Zambia DHS data from 1996 and 2007 (CSO *et al.*, 1997, 2009). Livingstone (urban site) and Kazungula (rural site) were selected as the two study sites.

Data were obtained from semi-structured, key informant interviews with officials in the MCDMCH (including some national-level officials in Lusaka), donor organizations (all in Lusaka), community-based organizations (CBO) involved in MNCH, and health care workers (HCW). Data were also obtained from four focus group discussions (two in Livingstone, two in Kazungula) with women who have had experience accessing MNCH services. Interviews and focus groups were conducted by one of the authors (MM) and two research assistants between August 20 and December 18, 2013.

#### Eligibility criteria and identification of study participants

All participants, whether key informants or focus group women, were eligible for the study if they met the following criteria: (1) being 18 years of age or older, (2) having adequate knowledge or experiences related to childhood survival specified for each participant group below, (3) speaking English, Tonga or Nyanja and (4) being able to provide written or verbal informed consent. Specific inclusion criteria for each key informant group included the following: national or provincial-level officials working in government-level health care system administration, policy-making, program development, leadership, or any aspect of MNCH (MCDMCH officials); directors, managers or other leaders of entities providing financial or other aid for MNCH services, or international or national organizations focusing on MNCH or with MNCH as one component of their mission (Donor organizations); directors, leaders, or managers working for a CBO involved in or providing referrals to MNCH services; and professionally trained physicians, nurses, clinical officers, or other health-related staff working in a health facility providing MNCH care (HCWs).

Similar numbers of participants from each key informant group were enrolled, and a range of ages, work experiences and positions/roles within each group was sought. Additionally, efforts were made to balance the number of urban and rural participants among the HCWs and CBO workers. Lists of potential key informants from each group were developed by the in-country research team with assistance, as needed, from the WHO National Programme Officer for Child and Adolescent Health and the MCDMCH Deputy Director for Child Health and Nutrition. A letter signed by an official from the MCDMCH was sent to each potential key informant participants informing them of the purpose of the study, risks and benefits of participation, and describing the interview process. These were followed-up with a phone call or email from the research team to set up a meeting time for those interested in participating. Among the MCDMCH (*n* = 6), CBO (*n* = 10) and HCW (*n* = 9) key informants, an equal number of men and women participated, while the donor organization participants (*n* = 6) were entirely female. Median ages were similar for the MCDMCH (47 years; Inter-quartile range [IQR]: 46–49), donor (44; 41–50), and CBO (46; 42–57) participants; HCWs were generally younger (41; 37–43). MCDMCH participants had spent a median of 21 years working in the Ministry (IQR: 16–25) compared to shorter durations spent with their respective organizations for donor (5 years; IQR: 4–12), CBO (7; 5–12), and HCW (4–14) participants.

Community women were recruited to participate in focus groups using informational flyers or advertisements. As with the key informants, a balance was sought in the level of education and the participants with live and deceased children, as well as a diversity of experiences and opinions regarding MNCH. Written informed consent was obtained from all participants who were enrolled. Rural (*n* = 21) and urban (*n* = 18) focus group participants had similar demographic and health characteristics except that rural women more often experienced the death of a child under 5 years old (33 vs 6%) (Table 2).

**Table 2.** Characteristics of female focus group participants in Zambia

	Rural participants (*N* = 21)		Urban participants (*N* = 18)	
Age, M (IQR)	26	(24, 36)	26	(21, 30)
Education, *N* (%)				
None	1	(5)	0	(0)
Primary	5	(24)	3	(17)
Secondary	15	(71)	13	(72)
Post-secondary	0	(0)	2	(11)
Travel time to health care (dry season), *N* (%)				
Less than 1 h	18	(86)	16	(94)
1–2 h	3	(14)	1	(6)
Number of living children, M (IQR)	2	(2, 5)	2	(1, 2)
Age of youngest child, M (IQR)	1 year	(1 year, 3 years)	1 year	(1 year, 3 years)
Any children who died <5 years old, *N* (%)				
No	14	(67)	17	(94)
Yes	7	(33)	1	(6)
Place of delivery for latest pregnancy, *N* (%)				
Health facility	21	(100)	17	(94)
Home	0	(0)	1	(6)
Birth attendant for latest pregnancy, *N* (%)				
Doctor	2	(10)	2	(11)
Nurse/midwife	17	(81)	15	(83)
Traditional birth attendant	1	(5)	0	(0)
Other	1	(5)	1	(6)

#### Interview and discussion guides

We developed interview guides for key informants and discussion guides for focus groups with community women. We then pilot tested them through cognitive interviewing, (Collins 2003) and revised as needed. The guides focus on barriers to and facilitators for improving child survival areas related to MNCH, corresponding to the structure for the review of national health policies and strategies (Table 1). Not all topics were appropriate for each key informant group, but each topic was asked of at least two of the four groups. While participants could discuss the entire period from 2000 forward, most participants recalled more recent information and experiences.

#### Data collection and analysis

Key informant interviews were conducted in English by one research assistant using the appropriate interview guide and were audio recorded. The focus group discussions were conducted in Tonga or Nyanja and also audio recorded. Two research assistants were present at each focus group to facilitate discussion and note-taking.

Following completion of the interviews and focus group discussions, audio recordings were transcribed by the research assistants, translated into English as needed, and field notes incorporated into the transcript. Transcripts were coded and analysed using the qualitative software Atlas.ti (ATLAS.ti Scientific Software Development GmbH, Berlin, Germany). Deductive themes were determined *a priori* based on interview guides and key topics of interest based on literature review. Additional themes were also identified upon review of the transcripts. Text was coded and reviewed for patterns of consistency, variation, relationships between themes and exemplary cases or quotations (Schensul 1993; Nastasi and Schensul 2005). Ethical approval for the project was obtained from Vanderbilt University Medical Center and ERES Converge (Zambia) Institutional Review Boards.

## Results

### MNCH coverage and other contextual indicators

**Figure 2.**
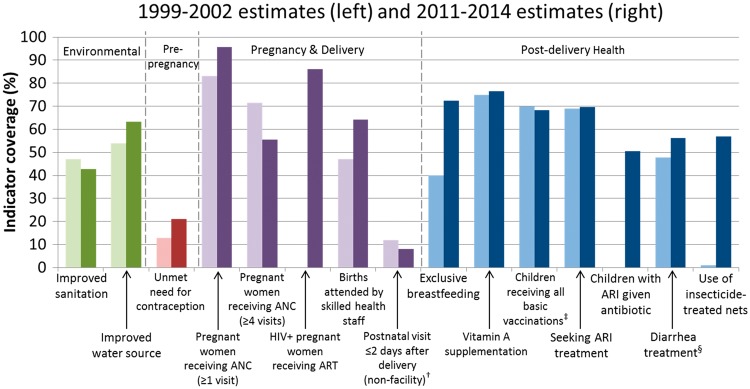
Changes in child survival indicator coverage in Zambia, 2000 and 2013*. *Estimates were not always available for years 2000 and 2013, in which case the nearest estimate between 1999 and 2002 or 2011 and 2014 was used; data were not available for the two indicators showing no coverage during the 2000 time period. ^†^Among births outside a health facility (excludes facility births). ^‡^Children 12–23 months old who have received BCG, measles and three doses each of DPT and polio vaccine (excluding polio vaccine given at birth). ^§^Children under 5 receiving oral rehydration and continued feeding Source for data: country DHS and the World Development Indicators Data Catalogue from the World Bank (accessed August 2015).

Zambia has improved coverage of 8 of the 13 core indicators shown in Figure 2 for which data were available for both 2000 and 2013. Coverage was the highest for women receiving ANC (96%), HIV-infected pregnant women receiving antiretroviral therapy (ART) for prevention of mother-to-child transmission (PMTCT) of HIV (86%), met need for contraception (79%), and vitamin A supplementation (77%) and exclusive breastfeeding (73%). Coverage was below 50% only for improved sanitation (43%) and postnatal visits within two days for non-facility deliveries (8%). Five indicators decreased during the study period. Notably, substantial declines were seen in the proportion of pregnant women receiving at least four ANC visits and met need for contraception which both declined substantially.

### Major themes from national documents, key informant interviews, and focus groups with community women

#### *Strong leadership, governance* *and political will to bring MNCH services to the community*

A major strength observed from review of national documents and also reported by key informants was the presence of strong political will and support for MNCH in Zambia, including policy, legislative, and strategic frameworks supporting the continuum from pregnancy through adolescence. Health sector reforms have been ongoing for over 20 years in Zambia, beginning with the first National Development Plan (NDP) in 1986. Subsequently, there have been successive, five-year NDPs (the 6^th^ NDP covered 2011–2015) and National Health Strategic Plans (NHSP; the 5^th^ NHSP covered 2011–2015). As such, strategies, plans, and guidelines are regularly revised as new research, treatment, and interventions become available. Key informants spoke further about the priority of bringing MNCH services closer to the community, reflecting the national health mission of providing ‘equity of access to effective quality health care as close to the family as possible’ (Zambia Ministry of Health [ZMOH], 2012b).

*There has always been this commitment [to] the child [which] is essential, is important, is vulnerable … the fact that within the ministry they make sure that child health receives attention … I think that commitment was very, very important and it works very well because it gives guidance.(44 year old, female donor partner)*

Consistent with this priority, Zambia has emphasized decentralized oversight and implementation of health services since the early 1990s. Key informants felt this was proving successful. They described how annual action plans and budgets continued to be developed at the facility and district levels, and were combined at the national level to ensure proper disbursement of funding to address needs at the local levels. This process facilitates implementation and scale up of needed MNCH services across all levels of the health system. Key informants also spoke about productive collaborations between different health programs, including leveraging available resources, whether funding, supplies or transportation, so that when one program helps another, both programs benefit. Bi-annual Child Health Weeks and use of CHWs, both described below, are examples of this successful implementation at the community level. Furthermore, there are structures in place to encourage stakeholders at the community and district level to participate via various committees, such as the Neighborhood Health Committees.

*We had a weak point with implementation at community level to allow community participation, but with … the creation of the new ministry [Ministry of Community Development, Mother and Child Health] we are strengthening and making sure that community linkages are always strengthened so that implementation is fully enhanced.(46 year old, male MCDMCH official)*

*I think because they [partners] are part of when the Ministry of Health is planning they call partners and we make commitments to their plan so through working with government and planning stage to actually meeting our obligations whether it’s provision of supplies, funding or hiring of consultants for analytical work, meeting the obligations in the plan that the government makes that’s how they’ve been very useful.(41 year old, female donor partner)*

In late 2011, the Ministry of Community Development and Social Services was realigned to include Mother and Child Health services (previously under the Ministry of Health) and re-named Ministry of Community Development, Mother and Child Health (MCDMCH). This was done to overcome implementation challenges identified during prior Health Strategic Plan periods, holistically address extreme poverty and primary health issues, and bring strong leadership and fresh impetus to MNCH programming. Key informants were excited about the realignment and some MCDMCH officials could already identify improvements in involving the community in planning and service delivery. (In September 2015, Zambia’s president discussed realigning maternal and child health back under the Ministry of Health, with the Ministry of Community Development remaining as such. As of August 2016, this has not yet occurred and maternal and child health remains within MCDMCH.)

Zambia has also taken an active approach to working with external partners to fund and carry out MNCH programs. Beginning in 2006 and continuing with the current NHSP, there has been a commitment to building partnerships, establishing memoranda of understanding, and leveraging these relationships to align partner activities with national MNCH strategies and plans. Key informants were generally positive about the role and contribution of external partners, and described how partners were involved in providing training, community education, personnel and materials to various MNCH programs.

*When these NGO’s they train volunteers on HIV prevention, then after training them they go to the community to give health education on HIV.(42 year old, male CBO partner)*

*[The] government has been complimented by partners resources … we have our own planning circle as government, so whichever activities are not covered by government, we lobby from partners to bridge the gap and that how we have been supplementing or mitigating this problem.(47 year old, female MCDMCH official)*

Some key informants expressed concerns about donors restricting the use of funds to specific areas or programs, or partners implementing their work in some areas but not others, leading to geographic disparities. But other key informants talked about how the MCDMCH works with partners in the context of national MNCH strategic plans to implement activities that supplement the work of the government and fill gaps where there are insufficient resources.

In line with its relatively strong health leadership, Zambia has also committed to funding MNCH. In 2011 Zambia reached the Abuja declaration target of 15% of government spending dedicated to health (UNAIDS, 2013). Key informants also felt that government funding had improved over time and that this increased funding reduced reliance on donor funding. With the exception of funding concerns for Child Health Weeks (discussed below), there was little discussion by key informants of insufficient funding, though some indicated that more funding would improve services and ensure sustainability as there continues to be reliance on external partners. In 2006 Zambia enacted a User Fee Removal policy ensuring free care for pregnant mothers and children under-five years in rural areas that was later expanded to include all pregnant mothers and children under-five years. Community women generally felt that services were available without much cost and only reported incurring small, nominal fees when seeking care.

*We have a lot of donor driven programs so we need more and more government driven funding for a lot of these programs to ensure that they actually continue and are sustainable. We are very happy that we have donors I am sure they will be with us for a while but … if we can reach a point where you don’t rely so much on external funding, then that is very good.(Female donor partner, age not reported)*

*An attempt has been [made by] the government to contribute to the health sector instead of entirely depending on the external funding … so there has been effort to fund the health sector. Although there is room for improvement.(51 year old, male MCDMCH official)*

*Even if you don’t have a husband you can still come to the clinic; you explain that you don’t have money [and] they can call an ambulance and it takes you to the general hospital.(36 year old, rural woman with 4 children)*

#### MNCH services delivered at the community level

Consistent with the priority to provide health services at the community level, a number of community-specific policies or strategies have been developed, including the Community Health Workers National Strategy (2009) (ZMOH, 2009), National Community Health Strategy (2010) (ZMOH, 2010), and most recently the National Community Health Assistant Program Implementation Guide (2012) (ZMOH, 2012a). Both the key informants and the community women spoke highly and regularly about the value of CHWs and other community-level health providers such as trained traditional birth attendants (TBA), traditional healers, and lay community members. Zambia has worked to formally involve these in the health system, training them to deliver in facilities, make referrals, or provide counselling and encouragement. While some key informants acknowledged *un*trained TBAs and healers could be a barrier to MNCH, community women were very knowledgeable about needing routine ANC and post-natal care and visits to under-five clinics for growth monitoring and immunizations, and recognized the important role of the trained TBAs and CHWs.

The CHWs are primarily responsible for educating the community about health issues, monitoring pregnant women and linking them to the appropriate services, and providing integrated packages of MNCH services through community Integrated Management of Childhood Illness (cIMCI) and integrated Community Case Management (iCCM). Key informants felt that the acceptability and use of many services, particularly ANC and immunizations, was attributable to the education and presence of the CHWs. Notably, no single MNCH intervention was identified as being under-implemented or not working well. Moreover, the high knowledge level of MNCH and what care was needed among community women reflects the effectiveness of these community-based efforts. Both key informants and the community women talked about the vital role CHWs have in linking community members with the health system and for encouraging utilization of recommended MNCH interventions.

*The community health workers come, they are being oriented, after orientation … they go in the community to sensitize others so that you can come at the clinic and bring their children when they are vaccinated. Then … the community are also aware of any diseases that are to come so that they can bring their children to the nearest clinic …(42 year old, male CBO partner)*

*They are those who are found in the community [community health workers] so you go to those to ask for help and they tell you what to do.(22 year old, urban woman with 1 child)*

Given the important role of CHWs and implementation of iCCM, it is important to note some of the challenges identified. First, some key informants felt that many CHWs still needed to be trained in iCCM. Because of this, iCCM has not been effectively implemented in all districts, particularly in areas with difficult terrain or sparse populations. While there was general agreement that more training was needed, key informants disagreed on whether funding for managing sick children at the community level was sufficient or not. A second concern expressed by some key informants was that CHWs are often volunteers (or receive small, non-monetary compensation or incentives). They suggested that regular pay or other forms of compensation would improve retention of CHWs. Last, some community women said they wanted more CHWs, which may be a reflection of a recent weakening in the CHW network that national documents suggested is being addressed.

Twice-annual Child Health Weeks were positively discussed by both key informants and community women. These intensified, facility-based and outreach efforts involve considerable effort and include CHWs, trained health care workers, and district-level officials. Child Health Weeks serve to bring preventive services such as immunizations and treated bednets closer to women and children in the communities where they are most needed. Key informants felt Child Health Weeks were good examples of collaboration and pooling of resources, but reported recent insufficient resources have threatened the sustainability of health services delivery. Women from one rural focus group further indicated that Child Health Weeks were no longer held in their area. These concerns may reflect the recent transition of funding from external partners to districts.

*If it is the week for child health week … they give the child polio [vaccination] for them to grow well … And you as a mother feel good that my child is growing well, that [the] medicine is working.(21 year old, urban woman with 1 child)*

*It is child health week, they have to go round in the community distributing the medicine but this time we didn’t see them.(24 year old, rural woman with 2 children)*

*In the last child health week which happened the community health workers were not well paid according … the number of days that they worked for [so that demoralized them].(41 year old, female HCW)*

Although access to community care has improved, community women, key informants and national documents all identified a lack of available facilities and transportation as major barriers to obtaining facility-based care. National documents outlined a government plan to build 650 health posts staffed by a new cadre of Community Heath Assistants (CHAs; described below) with support from donors and partners, and some women reported construction of new facilities was underway. Moreover, national documents, key informants, and community women regularly described shortages in HCWs, particularly among nurses and midwives and in rural areas. Key informants stated that most facilities have only one or two HCWs, and the community women stated they experienced long wait times at facilities and have occasionally been treated poorly or with disrespect by over-burdened HCWs. While these challenges made the visit unpleasant, it did not discourage them from accessing care. On the other hand, both national documents and qualitative study participants identified discomfort with being seen by male providers and shortages in medications at the health facility (except for HIV medication) as more significant deterrents.

*I brought my child and I found the nurse driving out. I asked are you not working at the clinic? [The nurse said] the clinic officer is not there so we cannot work that is how I went back with a sick child.(20 year old urban woman with 1 child)*

*It’s extremely varied, we know the understaffing is more hit in the rural areas where you may have one or two health workers for that facility doing everything and not just [MNCH] whether an adult comes with TB … has cardiac, whatever, so they have to take care of everything so the quality of service we can’t expect to be ideal.(41 year old, female donor partner)*

*Where there is a male nurse, male midwife, there is always shunning of the health services by our ladies in those communities … They feel uncomfortable to be attended to by a man and some of their husbands also feel it is not appropriate for their wives to be attended to by a male health worker.(43 year old, male HCW)*

National documents contained plans to address the HCW shortage by training direct entry midwives and by expanding the health workforce through task shifting to CHAs. The new CHA program is consistent with Zambia’s commitment to bring services as close to the community and family as possible, and also addresses concerns that existing CHWs are insufficiently trained and compensated. The CHAs receive more comprehensive and standardized training than CHWs, receive monthly pay for their work, and work with the existing CHWs. While it will take time before these new training programs reverse the current health care worker shortage, some key informants felt the shortage was already improving.

## Discussion

Zambia has successfully met MDG#4, reducing under-five deaths by two-thirds since 1990 to 64 deaths per 1,000 live births in 2015. This study sought to highlight some of the reasons for this success that can inform progress in child survival for other countries in the African Region in the post-2015 period. Strong leadership with a commitment to ongoing reform and efforts to ensure universal access to effective MNCH interventions have contributed to reducing under-five mortality in Zambia. By regularly reviewing and updating national health strategic plans, policies, and legislation, an environment has been created which prioritizes child health and promotes ongoing quality improvement and reform. More directly, Zambia has focused on bringing health services as close to the family as possible, as evidenced by specific community health strategies and implementation guides. This study suggests that CHWs have played an important role in providing basic child health education and services and linking women and children with the health system when other services are needed. Increased access to effective prevention and care has also been achieved though well-coordinated Child Health Weeks occurring twice each year that bring a full package MNCH services to the community. Zambia has not met its goal to improve MNCH without challenges, namely inadequate transportation, inconsistent availability of needed medication, workforce shortages, and insufficient financing. However, the country’s strong health leadership and commitment to ensure access to MNCH care down to the community level, combined with multi-sector collaborations and intentional partnerships with donors and other stakeholders, has enabled Zambia to successfully reduce under-five mortality and meet MDG#4 over the past two decades.

The important role of CHWs was highlighted in the Declaration of Alma Ata in 1978 when they were endorsed as key members of a primary health care team (Alma Ata 1978). There has been renewed interest in the utilization of CHWs over the past decade given the shortage of HCWs faced by many countries, most notably those in Africa (Haines *et al.* 2007). Additionally, studies have demonstrated that essential MNCH interventions and basic life-saving care can be provided by CHWs (Haines *et al.* 2007; Hermann *et al.* 2009; Yeboah-Antwi *et al.* 2010; Christopher *et al.* 2011 ). This study demonstrates that communities, health officials, and other stakeholders involved in MNCH have embraced CHWs as an integral and effective component of the Zambia’s care system. Several studies further describe the successful expansion of CHW skills to include community case management of pneumonia and malaria (Yeboah-Antwi *et al.*, 2010), the ‘teaming’ of CHWs and traditional birth attendants to provide better care across the continuum from newborns to under-fives (Yeboah-Antwi *et al.*, 2014), and training CHAs to fill in existing gaps in care (Zulu *et al.*, 2015).

Several challenges still must be overcome to maximize the potential of CHWs in Zambia. Training, financial compensation or other incentives have been established as important factors in retaining CHWs (Haines *et al.*, 2007, Alliance 2014, Campbell *et al.*, 2013). Zambia’s new national program for CHAs thus provides enhanced training, reimbursement and a positive work environment through pairing new and existing workers in the community they serve (ZMOH, 2012a). The CHAs provide an important link between the community and the health facility, ideally, spending 20% of their time at the health post and 80% of their time in the community alongside the CHWs (Zulu *et al.*, 2015).

There is ample evidence that national Child Health Weeks are a successful strategy for providing MNCH services as close to the family as possible. Notably, Zambia has remained one of the most experienced countries in conducting Child Health Weeks, far ahead of regional trends. This practice began in 1999 when Vitamin A supplementation was paired with the Supplemental National Immunization Days when only a few other countries were concurrently delivering multiple interventions (Fiedler *et al.* 2014). The proportion of African countries that have similarly increased the number of services delivered during Child Health Weeks to five or more has grown drastically from 8% in 2005 to 31% in 2010 (Palmer *et al.* 2013). One challenge that persists is the high level of external funding required to implement and sustain this strategy, with fluctuating funding year to year posing additional difficulty for planning and implementation (Fiedler *et al.* 2014). To address reliance on external funding, districts in Zambia have been directed to use their own resources for implementing Child Health Weeks, though women in our focus groups suggested that this transition may have temporarily disrupted provision of Child Health Weeks in some areas.

Finally, the essential role of external partners to ensure provision of effective MNCH services in Zambia cannot be overlooked. Their significant contribution has benefitted Child Health Weeks, provision of specific services such as HIV testing and treatment, training new cadres of health workers, on-the-job or specialty training for those already engaged in provision of MNCH services, and many others. In line with recommendations from the 2010 Global Strategy for Women’s and Children’s Health (United Nations 2010), the Zambian government has maintained up-to-date national health plans, making it easier to outline how partners can work within the existing system. Key informants did identify many areas where collaboration between MCDMCH and external partners could be improved, but the framework has been laid to ensure partners supplement the work of the MCDMCH and other Ministries by filling in gaps where there are insufficient resources.

The continued reliance on external partners and funding is a concern for sustainability, but Zambia has worked towards being more self-sufficient. The economy has been growing, with the gross domestic product (GDP) annual growth rate steadily increasing between 2000 and 2010 (World Bank), and in 2011 Zambia dedicated 15% of government spending to health, meeting the Abuja declaration target. Combined with strong political will, this made it possible for more financial resources to be available for MNCH and to accomplish the MNCH goals of the national health policy and national health strategic plan. However, the GDP annual growth rate has declined since 2011. Continued, strong political will is important to ensure ongoing progress in MNCH.

This study adds to the growing number of country case studies assessing progress towards MDG#4. We used a number of sources including review of national health policies and strategies across different sectors, qualitative data from key informants with different roles in MNCH, and four focus groups with community women from urban and rural areas. There are limitations for each of the study components. For the review of national MNCH policies and strategies, approximately one-third of the documents were 2013 drafts or revisions, even though the review was intended to cover the entire time period 2000–2013. More recent documents likely reflect newer strategies specifically developed to address many of the issues identified in the study, and may not fully explain why more rapid reductions in under-five mortality have occurred earlier in the study period. Moreover, country policies and strategies covered different and sometimes overlapping time periods, making it difficult to distinguish current from outdated information, and whether a stated plan had been implemented unless specifically stated in a document.

With regards to the interviews and focus groups, this study utilized a non-random sample of participants conducted in Livingstone, Kazangula and Lusaka. It is possible the views and experiences of some participants do not reflect those from other areas of Zambia. However, efforts were made to ensure results were reasonably representative of the larger population by involving individuals from areas that reflected national trends in under-five mortality as well as key informants with national-level responsibilities. In addition, although we asked participants to reflect on long-term changes, most of the participants recalled their current experiences and opinions on MNCH.

In spite of these limitations, this study provides important insight into Zambia’s steady progress in child survival. By bringing together diverse data sources, the study was able to assess existing activities and the degree of implementation and acceptability. The interviews and focus groups, in particular, provide important first-hand accounts of how MNCH-related strategies and programs are being realized in Zambia. The MNCH system has benefited from a steady series of health sector reforms that have emphasized decentralization and primary health care, including a strong community-based approach that actively engages the community in planning and decision-making. The collaboration between the Zambian government and external partners and funders has been positive, with the government slowly increasing its ability to fund and sustain key MNCH services.

To ensure further reduction in under-five mortality in the post-2015 era, Zambia will need to continue its progress towards health system strengthening to enhance MNCH care such as expanding the number of fully functional health facilities, training new cadres of midwives and community-level workers and bolstering partnerships to advance national child health priorities. Organizational and logistical challenges with integrating these newly trained HCWs into the existing health system still need to be addressed to ensure they reach their full potential. It is too early to know how the recent realignment of MCDMCH back into the Ministry of Health may affect this and other measures aimed at further reducing under-five mortality. Increased government funding of MNCH services can relieve reliance on external partners and ensure sustainability of the effective MNCH strategies that have enabled Zambia’s achievement of MDG#4.

## Supplementary Material

Supplementary Data
