# Supplementary material for: Improving access to child health services at the community level in Zambia: a country case study on progress in child survival, 2000–2013
Source: Health Policy Plan. 2016 Oct 19;32(5):603–12. doi: 10.1093/heapol/czw141 (PMC5964895; doi:10.1093/heapol/czw141)
Supplement: Supplementary Data [file czw141_supp.docx]

# SUPPLEMENTAL TABLE

## Table S1. Zambia policy, strategy, and other national documents reviewed

| **Document title (dates if not otherwise specified in title)** |
| --- |
| ***In-depth review* (Newest to oldest)*** |
| Strategic Plan for the Ministry of Community Development, Mother and Child Health 2013-2016 |
| Roadmap for Accelerating Reduction of Maternal, Newborn and Child Mortality 2013-2016 |
| Zambian National Health Policy, 2013 |
| Zambia Newborn Health Framework 2013 |
| National Health Strategic Plan, 2011-2015 |
| Sixth National development Plan, 2011-2015 |
| Republic of Zambia National Long Term Vision 2030 (2006) |
| National HIV/AIDS/STI/TB Policy (2005) |
| ***Brief review† (Newest to oldest)*** |
| National IMCI Strategic Plan 2013-17 (DRAFT) |
| Standard Treatment Guidelines, Essential Medicines List and Essential Laboratory Supplies List for Zambia (2013) |
| Zambia Essential Medicine list, 2013 |
| Immunization Vision & Strategy (2012) |
| Measles Campaign Report 2012 |
| Post-Measles Campaign Coverage Survey Report 2012 |
| National Community Health Assistant Program: Implementation Guide (2012) |
| Comprehensive Multi-Year Plan, 2011-2015 |
| National Food and Nutrition Strategic Plan for Zambia, 2011-2015 |
| Adolescent Health Strategic Plan 2011-2015 |
| National Community Health Worker Strategy in Zambia (2010) |
| Services Availability and Resource Assessment Report (2010) |
| Guidelines for Antiretroviral Therapy for HIV in Infants and Children in Zambia (2010) |
| Situational Analysis of Adolescent Health (2009) |
| Community Health Worker Strategy (2009) |
| National Nutrition Surveillance Survey Report (2008) |
| IMCI Health facility survey report for 2008 |
| Infant and Young Child Feeding Operational Strategy, 2006-2010 |
| National Food and Nutrition Policy (2006) |

*Primary documents extensively reviewed to obtain information on each content area identified in the abstraction guide

†Documents reviewed, but information on progress towards MDG#4 was sufficiently covered by the primary documents
